# ﻿Morphological and molecular studies of Mallomonas species from section Multisetigerae (Chrysophyceae, Synurales) with description of a new species

**DOI:** 10.3897/phytokeys.269.179128

**Published:** 2026-01-07

**Authors:** Evgeniy S. Gusev, Tatiana V. Safronova, Yulia А. Podunay, Hoan Tran, Alma B. Mohagan, Nikita A. Martynenko

**Affiliations:** 1 Severtsov Institute of Ecology and Evolution, Russian Academy of Sciences, Leninsky Prospect 33, 119071 Moscow, Russia Severtsov Institute of Ecology and Evolution, Russian Academy of Sciences Moscow Russia; 2 Komarov Botanical Institute of the Russian Academy of Sciences, Prof. Popova street 2, St. Petersburg, 197022, Russia Komarov Botanical Institute of the Russian Academy of Sciences St. Petersburg Russia; 3 T.I. Vyazemsky Karadag Scientific Station, Natural Reserve of the Russian Academy of Sciences, Nauki street, 24, Kurortnoe, Feodosiya, 298188, Russia T.I. Vyazemsky Karadag Scientific Station, Natural Reserve of the Russian Academy of Sciences Feodosiya Russia; 4 Joint Vietnam-Russia Tropical Science and Technology Research Center, 63 Nguyen Van Huyen, Nghia Do, Hanoi, 11307, Vietnam Joint Vietnam-Russia Tropical Science and Technology Research Center Hanoi Vietnam; 5 Central Mindanao University, Maramag, Bukidnon, Mindanao, Philippines Central Mindanao University Maramag Philippines

**Keywords:** *
Mallomonas
*, molecular phylogeny, new species, scale ultrastructure, tropics

## Abstract

This research is devoted to the study of Mallomonas species from the section Multisetigerae in a tropical region (Vietnam and the Philippines) using electron microscopy and molecular studies. For the first time, molecular data and phylogenetic relationships for species within this section are presented. Analysis of six algal cultures, based on a combined dataset of SSU rDNA and *rbc*L genes, as well as the ITS1–5.8S–ITS2 rDNA region, revealed three distinct clades. Notably, these clades did not form a monophyletic grouping. Two of the studied cultures were identified as *Mallomonas multisetigera*, and another two as *M. neoampla*. For the remaining clade, we describe a new species, *Mallomonas rutabuliformis***sp. nov.**, which combines morphological features of fossil and modern taxa. The study also synthesizes original and literature data on the distribution of the *Mallomonas multisetigera* morphotype.

## ﻿Introduction

Algae of the order Synurales represent a unique evolutionary lineage within chrysophytes, characterized by their intricate siliceous scales and bristles (Škaloud et al. 2013). These scales serve dual functions as structural supports that maintain cell morphology and as protective shields against environmental stressors (Siver 1991). Electron microscopy reveals that the ultrastructure of these scales and bristles exhibits remarkable species specificity, providing reliable diagnostic characters for taxonomic differentiation (Asmund and Kristiansen 1986; Kristiansen and Preisig 2007; Škaloud et al. 2013). The scale architecture demonstrates particular complexity, featuring multiple specialized structures. Many species possess ribs, reticulations, and papillae, along with distinctive anterior submarginal ribs and V-ribs. The V-rib forms a conspicuous V-shaped siliceous ridge, with its base anchored in the proximal scale region and its arms extending to the mid-scale. Typically, two anterior submarginal ribs emerge near the end of the V-rib, running parallel to the distal margins before terminating near the scale apex (Siver 1991). Additional structural elements include papillae, struts, ribs, and pores of different diameters and structures. The specific configuration, spatial organization, and dimensions of these features represent taxon-specific diagnostic markers that show congruence with molecular phylogenetic data (Jo et al. 2011; Siver et al. 2015). Complementing the scale covering, most *Mallomonas* species bear siliceous bristles—specialized elongated structures firmly anchored to specific scales. Bristles are elongated structures, each composed of a flat basal portion called the foot and a long, slender shaft (Siver 1991). For species with domed scales, the foot is fitted within the cavity of the dome, and the shaft emerges from an inverted U-shaped opening, allowing the bristle to rotate relative to the swimming axis. Like the scale, the structure and distribution of bristles on the cell are of taxonomic significance (Siver and Lott 2012).

The good preservation of scales, bristles, and cysts enables reconstruction of the evolutionary history of synuralean algae. Key studies on this topic have been published within the last 20 years based on material from the Giraffe Pipe locality in Canada (Siver and Wolfe 2005; Siver et al. 2009; Siver et al. 2015). The oldest records of *Mallomonas* scales and bristles found so far are from middle Eocene deposits (Siver and Wolfe 2005). Scales and bristles representing numerous species have been identified, including taxa that are surprisingly similar in structure to modern species (Siver and Lott 2012; Siver 2023), as well as others with unique morphological characters that are presumed to be extinct (Siver and Wolfe 2010; Siver 2023). Many of the extinct species possessed large and robust scales, often four times larger than those of most modern species (Siver et al. 2015, 2022). Based on model estimates, the mean cell size of the fossil species is approximately twice as large as the average cell size of modern organisms (Siver 2022).

One of the diagnostic features of fossil *Mallomonas* species is the presence of domes that are recessed from the scale margin. These domes typically exhibit an elongated oval shape, enabling the anchoring of multiple bristles within a single dome. At least three Eocene *Mallomonas* taxa from different sections possess such recessed domes: *M. preisigii* Siver (section Papillosae), *M. ampla* Siver and Lott (section Multisetigerae), and *M. media* Siver and Lott (section *Planae*) (Siver and Lott 2012). The domes may vary in prominence, ranging from shallow and weakly defined, e.g., *M. preisigii*, to large and conspicuous, e.g., *M. ampla* (Siver and Lott 2012). The bristles of fossil taxa are characterized by a distinctly hooked foot (Siver et al. 2009, 2015, Siver and Lott 2012). Recent discoveries of tropical species—*M. vietnamica* Gusev, Kezlya and Tran, *M. neoampla* Gusev and Siver, and *M. acidophila* Gusev and Shkurina from Vietnam—reveal that their ultrastructural elements closely resemble those of fossil scales, suggesting the persistence of relict taxa in modern flora (Gusev and Siver 2017; Gusev et al. 2021a, 2023a).

Within the group of species exhibiting ancient morphological features, morphotypes resembling *Mallomonas multisetigera* Dürrschmidt (section Multisetigerae) are particularly noteworthy. This group includes the widely distributed modern species *M. multisetigera*, the fossil taxon *M. ampla*, and *M. neoampla*—a transitional species displaying characteristics of both fossil and modern taxa. However, molecular data for section Multisetigerae remain unavailable to date. Furthermore, our research revealed cultures of an additional novel taxon belonging to this group.

The objectives of this study are to determine the phylogenetic relationships of species within section Multisetigerae and to characterize a new species that bridges morphological features between fossil and modern taxa.

## ﻿Materials and methods

### ﻿Study area

Water samples from four localities in four provinces in Vietnam were included in this study (Table 1). Samples were collected during expeditions of the Joint Vietnam–Russia Tropical Science and Technology Research Center (the “Ecolan 3.2” project) conducted between 2014 and 2020. Descriptions of the climatic and geographical features of the provinces are provided in previously published studies (Gusev et al. 2017, 2019, 2021b; Tran and Mazei 2018; Tran et al. 2022). In general, this area has a tropical monsoon climate with high annual precipitation that varies in timing and amount between provinces and high relative humidity (Schmidt-Thomé et al. 2015). In addition, material from Mindanao Island, Philippines, was included in the study (Table 2).

**Table 1.** List of studied cultures with information about localities in Vietnam, environmental parameters, and GenBank accession numbers (Cond.—specific conductance, µS cm^-1^; T—temperature, °C; SSU+ITS, *rbc*L—GenBank accession numbers).

Culture (Identification)	Locality	GPS	pH	Cond.	T	SSU+ITS	*rbc*L
**Thanh Hoa Province**							
277Yu *M. neoampla*	Pool in rice field	19°44.108'N, 105°44.496'E	7.0	263	31	PX698599	PX703665
**Khanh Hoa Province**							
VN819 *M. neoampla*	Dzua River	12°15.017'N, 109°09.083'E	6.6	246	33	PX698598	PX703664
**Lam Dong Province**							
VNG2139 *M. rutabuliformis*	Stream in the forest	12°10.540'N, 108°41.996'E	7.7	18	19	PX698596	PX703662
VNG2127 *M. rutabuliformis*	Stream in the forest	12°10.540'N, 108°41.996'E	7.7	18	19	PX698597	PX703663
**Dong Nai Province**							
VNG2052 *M. multisetigera*	Dak Lua swamp, Cat Tien National Park	11°31.023'N, 107°23.289'E	5.5	47	32	PX698594	PX703660
VNG2053 *M. multisetigera*	Dak Lua swamp, Cat Tien National Park	11°31.023'N, 107°23.289'E	5.5	47	32	PX698595	PX703661

**Table 2.** Basic characteristics of the studied localities in the Philippines (GPS—coordinates, Cond.—specific conductance, µS/cm; T—temperature, °C).

No.	Localities	GPS	pH	Cond	T
1	Lake Pinamaloy, Barangay Pinamaloy, Don Carlos, Bukidnon	7°40.436'N; 125°00.063'E	6.9	53	29
2	Lake Pinamaloy, Barangay Pinamaloy, Don Carlos, Bukidnon	7°40.421'N; 125°00.141'E	6.6	49	31
3	Opalon Spring, Barangay Butong, Quezon, Bukidnon	7°47.807'N; 125°03.984'E	7.1	202	27
4	Opalon Stream, Barangay Butong, Quezon, Bukidnon	7°47.777'N; 125°03.989'E	7.1	200	27
5	Opalon Swamp, Barangay Butong, Quezon, Bukidnon	7°47.781'N; 125°03.979'E	7.4	155	39
6	Forest waterbody in Barangay Lunotan, Gingoog City, Misamis Oriental	8°42.054'N; 125°01.044'E	5.5	14	24
7	Water pool 1 near pond in Barangay Lunotan, Gingoog City, Misamis Oriental	8°42.263'N; 125°00.923'E	5.2	13	22

### ﻿Samples and collections

Planktonic samples were collected using a plankton net with a 20 μm mesh size for culture isolation. Water mineralization and temperature were measured using a Hanna device (HI 9828, Hanna Instruments, Inc., Woonsocket, RI, USA). Cultures were isolated by E.S. Gusev, N.A. Martynenko, and Yu.A. Podunay. Cultures were permanently deposited in the Collection of the Severtsov Institute of Ecology and Evolution, Russian Academy of Sciences.

### ﻿Culturing

Monoclonal cultures were established by examination of micropipetted single cells under an inverted microscope. Non-axenic unialgal cultures were maintained in modified WC, DY-V, and Waris-H liquid culture media (McFadden and Melkonian 1986; Andersen 2005) at 22 °C in a growth chamber with a 12:12 h light:dark photoperiod and a light intensity of 50–100 µmol m^−2^ s^−1^. In total, six cultures were isolated from different regions of Vietnam and used for phylogenetic analyses based on SSU rDNA + *rbc*L cpDNA and ITS1–5.8S–ITS2 rDNA datasets.

### ﻿Extraction of DNA and amplification

Total DNA from monoclonal cultures was extracted using InstaGeneTM Matrix according to the manufacturer’s protocol. Fragments of partial SSU rRNA (1713 bp) were amplified using the primer pairs 18S-F (Katana et al. 2001) and 18L (Hamby et al. 1988). For ITS1–5.8S–ITS2 rRNA (604–627 bp), the primer pair KN1 (Wee et al. 2001) and Chryso_ITSR (Škaloud et al. 2012) was used. Amplification of the *rbc*L cpDNA marker (654 bp) was performed using the primers *rbc*L_2F (Daugbjerg and Andersen 1997) and Synura_rbcLR (Gusev et al. 2018). Amplification of all fragments was carried out using the premade ScreenMix (Evrogen, Russia) for polymerase chain reaction (PCR). Amplification conditions for partial rDNA fragments included an initial denaturation of 5 min at 95 °C, followed by 35 cycles of denaturation at 94 °C for 30 s, annealing at 52 °C for 30 s, extension at 72 °C for 40–90 s, and a final extension of 10 min at 72 °C. Amplification conditions for the *rbc*L fragments were the same, except for the number of cycles (40) and the annealing temperature (48 °C). Resulting amplicons were visualized by horizontal agarose gel electrophoresis (1.5%) and stained with SYBR Safe (Life Technologies, Carlsbad, CA, USA). DNA fragments were purified using the ExoSAP-IT kit (Affymetrix, Santa Clara, CA, USA) according to the manufacturer’s protocol. All fragments were sequenced using forward and reverse PCR primers and the BigDye system (Applied Biosystems, Foster City, CA, USA), followed by electrophoresis on a Genetic Analyzer 3500 sequencer (Applied Biosystems, Foster City, CA, USA). In addition, SSU rDNA fragments were sequenced using the internal primers 18S-826F (Choi et al. 2013) and picoR2 (Belevich et al. 2015) to assemble and verify the resulting sequences. Sequences were checked manually and assembled using MegaX (Kumar et al. 2018).

### ﻿Alignment and phylogenetic analysis

Newly determined sequences and GenBank sequences of 68 other *Mallomonas* cultures were included in the alignment. In addition, the synurophycean taxa *Synura americana* Kynclová and Škaloud, *Synura mammillosa* E. Takahashi, and *Neotessella lapponica* (Skuja) B.Y. Jo, J.I. Kim, W. Shin, P. Škaloud and P.A. Siver were added to the dataset as outgroup taxa. The sequences were aligned using either the global SILVA alignment in SINA v1.2.11 (Pruesse et al. 2012) for SSU rDNA or MAFFT v7 with the auto strategy (Katoh et al. 2019) for *rbc*L cpDNA and ITS1–5.8S–ITS2 rDNA gene fragments. Two separate phylogenetic analyses were performed: one based on concatenated partial SSU rDNA + *rbc*L cpDNA fragments and the other using ITS1–5.8S–ITS2 rDNA sequences. The resulting SSU rDNA + *rbc*L cpDNA dataset (2367 bp) was partitioned into different genetic regions, and the most appropriate substitution model for each partition was estimated separately using the Bayesian information criterion (BIC) in jModelTest 2.1.10 (Darriba et al. 2012). The selected best-fit model for SSU rDNA was GTR + G + I. For each codon position of the protein-coding *rbc*L cpDNA gene, the best model was also tested. The BIC-based model selection procedure identified the following models: GTR + G + I for the first codon position, JC + I for the second codon position, and GTR + G for the third codon position. Bayesian inference (BI) analysis was conducted using MrBayes 3.2.5 (Ronquist and Huelsenbeck 2003). Three hot and one cold Markov chains were run for 15 × 10^6^ cycles in two independent runs, sampling every 100^th^ generated tree. Phylogenetic trees and posterior branching probabilities were obtained after discarding the first 25% of trees as burn-in to estimate parameters of nucleotide substitution models and likelihood.

The dataset based on ITS1–5.8S–ITS2 rDNA sequences was constructed using nine *Mallomonas* cultures, including cultures of *M. vietnamica*, *M. multisetigera* (PV036658), and *Mallomonas ouradion* K. Harris and D.E. Bradley (PV036660), and contained 651 aligned positions. For both the SSU rDNA + *rbc*L and ITS datasets, maximum likelihood phylogenies (ML) were constructed using IQ-TREE (Chernomor et al. 2016) with the models and partitions described above. Bootstrap analyses were performed with 1,000 replicates. Viewing and editing of trees were carried out using FigTree v1.4.2 and Adobe Photoshop CC (19.0).

### ﻿Electron microscopy

For electron microscopy studies, an aliquot of each sample was washed three times by repeated centrifugation with deionized water. Drops of each washed sample were dried directly onto stubs for scanning electron microscopy (SEM) or onto grids for transmission electron microscopy (TEM) or digested for 4–5 min in sulfuric acid with potassium dichromate before mounting. For SEM studies, samples were dried onto aluminum stubs, coated with gold for 10 min using a Q150R ES Plus (Quorum Technologies Ltd., East Sussex, UK), and observed with a TESCAN MIRA 3 LMH (TESCAN, Brno, Czech Republic). For TEM studies, samples were dried onto formvar-coated grids (EMS FF200-Cu-50, Electron Microscopy Sciences) and observed using a JEM-1011 and Hitachi H-600 TEM. Specific conductance, pH, and temperature measurements were made using a Hanna HI 9828 device (Hanna Instruments Inc., USA).

## ﻿Results

**Figure 1.**
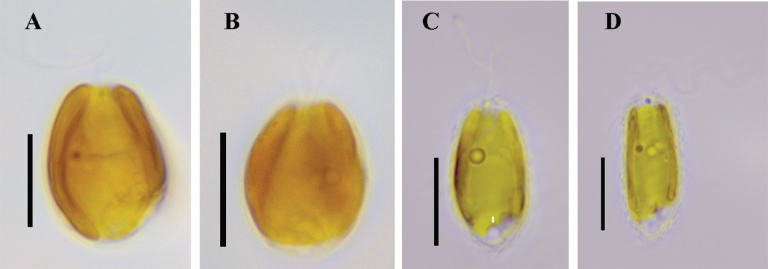
Light micrographs of cells of *Mallomonas rutabuliformis* sp. nov. and *M. multisetigera* Dürrschmidt. **A, B.***M. rutabuliformis*, culture VNG2139; **C, D.***M. multisetigera*, culture VNG2052. Scale bars: 10 µm.

**Figure 2.**
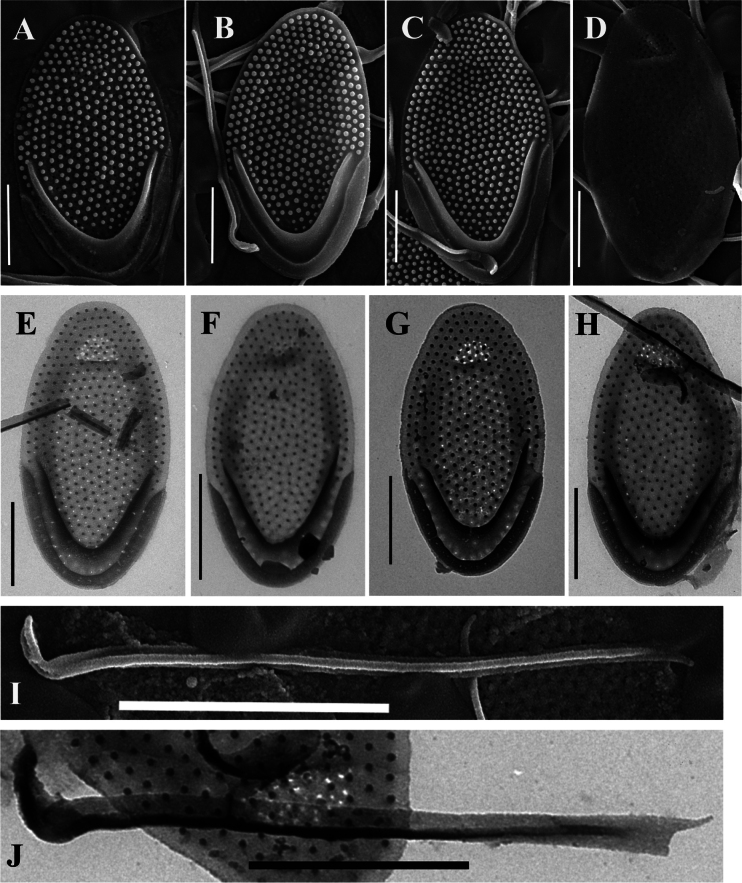
*Mallomonas rutabuliformis* sp. nov. (authentic culture VNG2139), studied using TEM and SEM. **A–C.** Body scales, note regularly arranged papillae on the shield and on the dome, SEM; **D.** Undersurface view of the scale showing a group of pores in the central part and in the dome, SEM; **E–H.** Body scales, note the prominent and rounded V-rib and posterior flange with few minute base plate pores, TEM; **I.** Bristle with a hook-shaped flattened foot, SEM; **J.** Bristle with a hook-shaped flattened foot, TEM. Scale bars: 1 µm (**A–H**); 2 µm (**I, J**).

**Figure 3.**
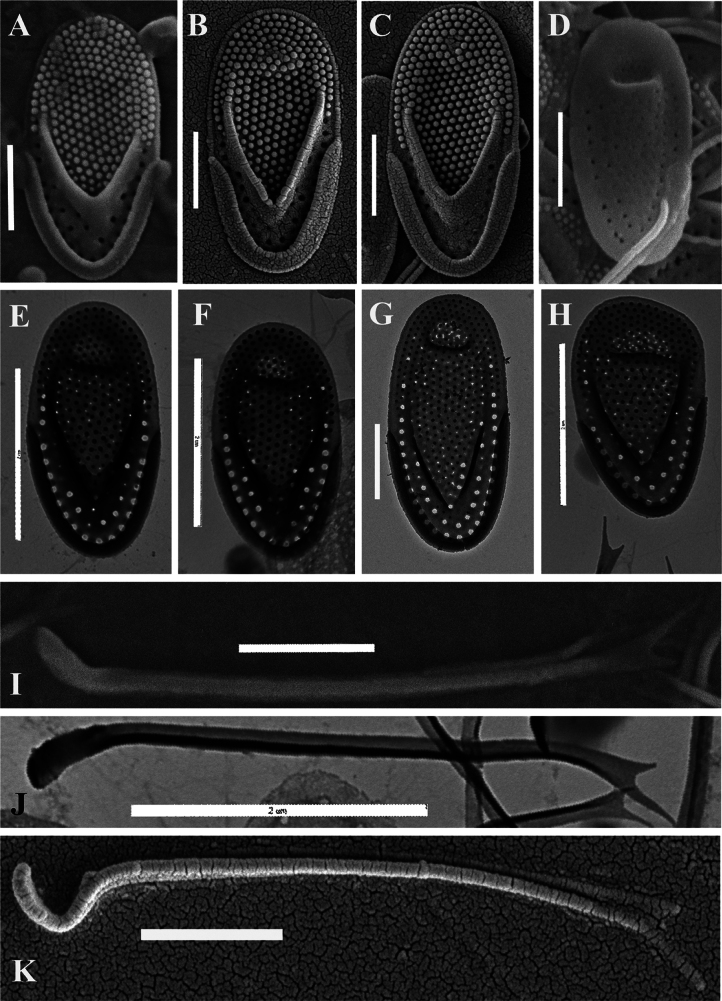
*Mallomonas multisetigera* Dürrschmidt (culture VNG2052), studied using TEM and SEM. **A–C.** Body scales, note regularly arranged papillae on the shield and on the dome, SEM; **D.** Undersurface view of the scale showing a group of pores in the central part and on the posterior flange, SEM; **E–G.** Body scales, note the conspicuous and acute V-rib and posterior flange with large base plate pores, TEM; **H.** Asymmetric anterior scale, TEM; **I.** Bristle with a flattened foot, turned at an angle of approximately 45°, SEM; **J.** Bristle with a flattened foot, turned at an angle of approximately 45°, TEM; **K.** Bristle with a hook-shaped base, SEM. Scale bars:1 µm (**A–D, G, I, K**); 2 µm (**E, F, H, J**).

**Figure 4.**
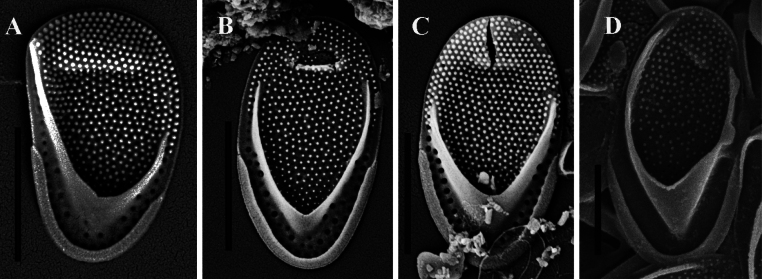
*Mallomonas multisetigera* Dürrschmidt, discovered in the Philippines (**A–C**), and *M. neoampla* (culture 277Yu) from Vietnam (**D**), studied using SEM. **A.** Asymmetric anterior scale, *M. multisetigera*; **B, C.** Body scales, *M. multisetigera*; **D.** Body scale, *M. neoampla*. Scale bars: 2 µm.

**Figure 5.**
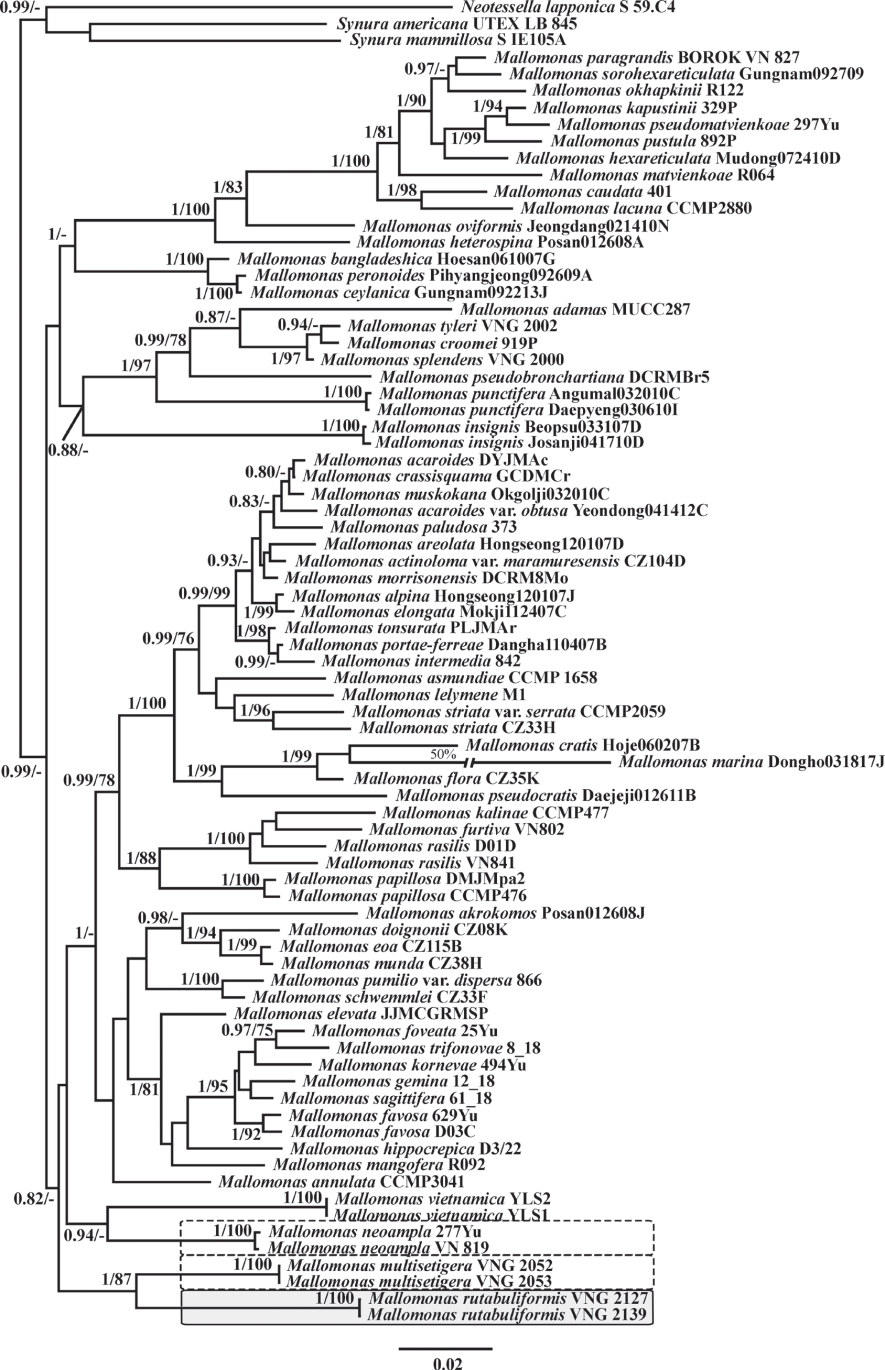
Bayesian consensus tree of the nuclear small subunit rDNA (SSU rDNA) and chloroplast *rbc*L concatenated dataset. The Bayesian posterior probability (> 0.80) and maximum likelihood bootstrap value (> 70%) are shown to the left and right of the fraction line, respectively. Scale bar represents substitutions per site. *Mallomonas rutabuliformis* sp. nov. is marked with a solid-line box, *M. neoampla* and *M. multisetigera* with dashed rectangular boxes.

**Figure 6.**
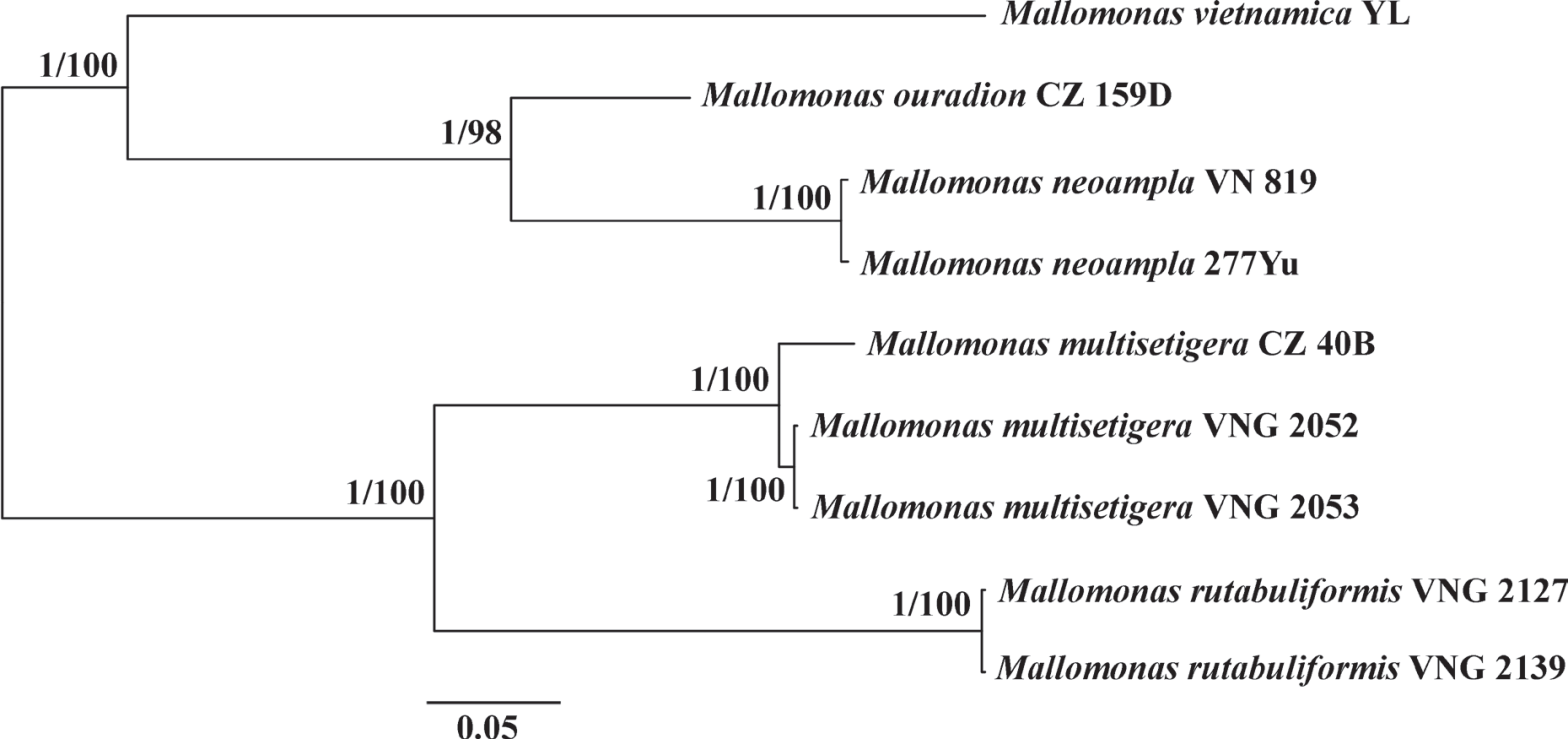
Unrooted Bayesian tree based on the ITS1–5.8S–ITS2 sequences of selected *Mallomonas* species. The Bayesian posterior probability (> 0.80) and maximum likelihood bootstrap value (> 70%) are shown to the left and right of the fraction line, respectively. Scale bar represents substitutions per site.

In total, four new cultures similar in morphology to *Mallomonas multisetigera* and two representing *M. neoampla* were revealed from four localities during studies of freshwater chrysophytes in Vietnam (Table 1, Figs 1–4). Phylogenetic relationships inferred using maximum likelihood (ML) and Bayesian inference (BI) from datasets of nuclear-encoded SSU rDNA and plastid-encoded *rbc*L, studied for six representative cultures of section Multisetigerae, showed that the organisms were divided into three clades (Figs 5, 6). Notably, these clades did not form a monophyletic grouping. The clade comprising cultures VNG2052 and VNG2053 was attributed to *Mallomonas multisetigera*. The clade comprising cultures VN819 and 277Yu represented *M. neoampla*. For the clade comprising cultures VNG2139 and VNG2127, we propose a new species.

Below, we describe the new species based on molecular and morphological data and provide an expanded description of the *Mallomonas multisetigera* culture.

### ﻿ Mallomonas rutabuliformis

Taxon classificationPlantaeSynuralesMallomonadaceae

E.S. Gusev, Safronova, Podunay & Martynenko sp. nov.

4B0E77A1-11A8-5D18-91E8-F9E2C8317117

Figs 1A, B, 2

#### Description.

Cells oval or ovate, 8.3–15.9 × 6.6–11.2 μm, covered by scales with bristles. Scales oval or elongated oval, 3.2–4.1 × 1.8–2.2 μm, with a dome. Shield with regularly arranged papillae and minute base plate pores (approx. 0.01–0.03 μm). The dome is shallow, broadly oval or rounded, recessed from the anterior margin, and is covered with papillae. In some cases, the dome is perforated with pores. The anterior flange is slightly raised above the shield, with two or three rows of papillae and few minute base plate pores. The V-rib is prominent and rounded, with arms of unequal length. The posterior rim is narrow, encircles approximately one-third of the scale perimeter. The posterior flange is narrow, with few base plate pores. Bristles are 2.3–6.2 μm long, slightly curved, with a hook-shaped flattened foot, turned at an angle from 45 to 90 degrees relative to the shaft. The margins of the bristles are rolled to form an open U-shaped groove, the distal tip is bifurcated, with spines of unequal length. Cysts were not observed.

#### Holotype.

(here designated): Portion of a single gathering of cells on SEM stub, deposited at the Herbarium, Komarov Botanical Institute RAS (LE), Saint Petersburg, Russia (LE A0007759). Material from a stream in the forest in Lam Dong Province, Vietnam, leg. N.A. Martynenko, 8 June 2019. Fig. 2A is a representative scale from the specimen.

#### Type locality.

Vietnam, Lam Dong Province, stream in the forest. Latitude/longitude: 12°10.540'N, 108°41.996'E.

#### Reference culture.

Culture VNG2139: Representative living and fixed culture VNG2139 and DNA sample deposited at the Collection of the A.N. Severtsov Institute of Ecology and Evolution, Russian Academy of Sciences.

**Genbank accession number for reference culture (VNG2139)**: PX698596 (nuclear SSU+ITS rDNA) and PX703662 (*rbc*L cpDNA).

#### Etymology.

The epithet is named based on the morphological character of the bristles, which are bent in the distal part, reminiscent of a poker shape.

#### Distribution.

Species was found only in the type locality.

#### Observations.

*Mallomonas rutabuliformis* was found at pH 7.7, a specific conductance of 18 μS cm^-1^ and temperature 19 °C (Table 1).

##### ﻿Description of *Mallomonas multisetigera* culture

In our studies, we found morphotype of *Mallomonas multisetigera*, similar in ultrastructure to originally described form South Chile (Dürrschmidt 1982). Here, we provide a description of our cultures based on LM, SEM, and TEM studies and complement them with molecular studies.

### ﻿ Mallomonas multisetigera

Taxon classificationPlantaeSynuralesMallomonadaceae

Dürrschmidt

E7175DE6-F677-5F7D-93F2-C201B69589F0

Figs 1C, D, 3

#### Description.

Cells ellipsoidal, 8.6–13.5 × 3.7–7.9 μm, covered by scales with bristles. Body scales oval, 2.3–3.5 × 1.2–1.7 µm, tripartite. Shield with regularly arranged papillae and scattered minute base plate pores (0.01–0.03 μm). Sometimes larger pores can be observed on the shield. The dome is shallow, broadly oval, set back from the anterior margin, and covered with papillae. The V-rib is conspicuous, acute, and hooded. The posterior rim is narrow, encircles less than a half of the scale perimeter. The posterior flange is wide, with two rows of large base plate pores (0.03–0.08 μm). The anterior flange with two or three rows of papillae and often with a few large base plate pores continuing from the posterior flange. Anterior scales are asymmetric, widened in the distal part, with elongated dome and have the same basic structure as body scales. Bristles are 2.6–5.0 µm. The foot of the bristle is flattened, turned at an angle of approximately 45 degrees or less relative to the shaft, the margins of the bristles are rolled to form an open U-shaped groove, the distal tip is bifurcated, with spines of unequal length. Cysts were not observed.

#### Observations.

*Mallomonas multisetigera* was found at wide ranges of environmental parameters: pH from 4.7 to 7.2, specific conductance from 25 to 2730 µS cm^-1^, and temperature 29–39 °C (Table 1, and see Gusev et al. 2023b).

## ﻿Discussion

Based on scale ultrastructural characteristics, all examined species were assigned to section Multisetigerae. This section was originally established in 1986 and initially comprised only a single species, *Mallomonas multisetigera* (Asmund and Kristiansen 1986). First described from southern Chile in 1982 (Dürrschmidt 1982), *M. multisetigera* exhibits several distinctive morphological features. Its scales are tripartite, featuring a well-defined V-rib whose arms curve. The scales also possess a shallow, broad dome that is recessed from the anterior margin and numerous small papillae densely covering the shield, dome, and often the anterior flanges. Notably, the base plate pores are significantly larger on the posterior flange compared to the rest of the scale. Another unique characteristic of this species is the frequent association of scales with multiple bristles, with up to five bristles reported on anterior scales (Dürrschmidt 1982). Subsequently, the species has been frequently recorded in various regions worldwide, showing considerable variability in scale ultrastructure. This species is considered a widely distributed taxon (Kristiansen and Preisig 2007). In Europe, it has been recorded in Denmark (Kristiansen 1978), the Netherlands (Roijackers and Kessels 1986), Finland (Hällfors and Hällfors 1988), the Czech Republic (Pichrtová and Vesela 2009; Němcová 2010), France (Němcová et al. 2012), Austria (Pichrtová et al. 2013), Sweden (Němcová et al. 2016), and Russia (Kotkova et al. 2025). Outside Europe, it is known from Japan (Takahashi 1978, reported as sp. No. 25 in Asmund and Kristiansen 1986), Jamaica (Cronberg 1989), Madagascar (Hansen 1996), Nigeria (Wujek et al. 2003, 2010), Brazil (Franceschini and Kristiansen 2004; Wujek and Bicudo 2004), Indonesia (Gusev et al. 2022a, b), Ecuador (Wujek and Dziedic 2005), and Vietnam (Gusev et al. 2023b).

To assess the variability of the body scales of *M. multisetigera*, we compared the type material from Chile (Dürrschmidt 1982) with scales from culture VNG2052 of *M. multisetigera* and with published data from other geographical locations. *Mallomonas multisetigera* is a widespread species found on almost all continents (Table 3). The variability among modern findings is primarily expressed in differences in the size of the body scales and the number and arrangement of large pores. In the original description (Dürrschmidt 1982), the number of large pores on the posterior flange of the body scales was reported as 45–51, arranged in two rows. The number of large pores on the shield varies from 10 (Dürrschmidt 1982: fig. 8) to more than 90 (Dürrschmidt 1982: fig. 9).

**Table 3.** Some morphological features of scales of different populations of *Mallomonas multisetigera*.

Reference	Length, μm	Width, μm	Number of pores on the posterior flange	Number of pores on the shield	Length: width ratio
Dürrschmidt 1982 (Chile)	3.5–3.6	2–2.1	53–54	9–93	1.65–1.71
Wujek and Bicudo 2004 (Brazil)	3.4–3.7	1.9–2.1	18–20	2	1.65–1.94
Franceschini and Kristiansen 2004 (Brazil)	4–4.5	2.3	18–20	9, 8, 57	1.76–1.91
Wujek and Dziedic 2005 (Ecuador)	4.1	2.4	61	39	1.7
Carty and Wujek 2003 (Belize)	4	2	57	79	1.94
Cronberg 1989 (Jamaica)	5.1–5.3	2.4–2.7	39–57	–	1.89–2.02
Němcová et al. 2012 (France)	3	1.6	54	63	1.88
Pichrtová and Vesela 2009 (Czech Republic)	4.1	2.3	58	46	1.78
Kotkova et al. 2025 (Russia)	3.6	2	70	99	1.8
Hansen 1996 (Madagascar)	4.6	2.3	66	70	1.81–1.88
Philippines, this study	4–4.3	2.2–2.3	53–66	16–20	1.80–1.84
Culture VNG2052, this study	2.3–3.5	1.2–1.7	19–39	2–4	1.81–2.0

In South America, besides the type locality, the species has been found in Brazil (Franceschini and Kristiansen 2004; Wujek and Bicudo 2004). However, the number of large pores on the posterior flange of the body scales is almost three times lower (16–21) than in the original description, and large pores on the shield are either almost absent or significantly fewer in number, ranging from 2–5 to 56. A distinctive feature of the Brazilian specimens is the arrangement of pores on the posterior flange in a single row (Franceschini and Kristiansen 2004: figs 7–9; Wujek and Bicudo 2004: fig. 1).

The findings of *M. multisetigera* from Europe are most similar to the original description in terms of the number and arrangement of large pores. However, since the micrographs provided in publications do not always allow for an accurate count of the large pores, we considered the following European records: Finland (Hällfors and Hällfors 1988), the Czech Republic (Pichrtová and Vesela 2009), France (Němcová et al. 2012), Austria (Pichrtová et al. 2013), and Russia (Kotkova et al. 2025). In Europe, the number of large pores on the posterior flange ranges from 43 to 61, whereas on the shield it varies from 46 (Czech Republic, Pichrtová and Vesela 2009) to approximately 100 (Russia, Kotkova et al. 2025).

The *M. multisetigera* culture VNG2052 corresponded to the original description by Dürrschmidt (1982) both in scale dimensions (body scales 2.5–5 × 1.8–2.8 µm from the type habitat versus 2.3–3.5 × 1.2–1.7 µm in culture VNG2052) and in ultrastructural details. The only distinct difference in the Vietnamese organism was the low number of large pores on the shield. However, as demonstrated above, this is a variable character.

ITS1–5.8S–ITS2 rDNA analysis revealed genetic differences between the European (culture CZ_40B) and Vietnamese populations (cultures VNG2052 and VNG2053), but they were not substantial enough (18 nucleotides and 3.5%) to confidently conclude that they represent different species (Fig. 6). Further research and the study of more populations from different geographical regions are required to clarify the taxonomy of this species. For now, we suggest that *M. multisetigera* is a widely distributed species with considerable morphological variability.

The second species from this section, *Mallomonas ampla*, was described in 2012 from middle Eocene mudstones at the Giraffe locality situated near the Arctic Circle in northern Canada (Siver and Lott 2012). Scales of this fossil species, which are significantly larger than those of *M. multisetigera*, have base plate pores with uniform diameter over the entire surface and a dome recessed from the anterior margin. In addition, bristles of *M. ampla* are distinctly recurved and hook-like at the proximal end. Given similarities in scale ultrastructure, especially in dome structure, between *M. ampla* and *M. multisetigera*, Siver and Lott (2012) suggested that the lineage represented by section Multisetigerae is ancient, extending to at least the Eocene and likely considerably further back in geologic time.

The third species from section Multisetigerae, *M. neoampla*, was described recently from Vietnam (Gusev and Siver 2017). This species shares morphological characters with both *M. multisetigera* and *M. ampla*. Scales of *M. neoampla* and *M. ampla* are of similar size and are both significantly larger than those of *M. multisetigera*. The bristles of both species are also similar in structure. On the other hand, *M. neoampla* and *M. multisetigera* share similar scale morphology. The degree to which the dome is recessed from the anterior margin is greatest in *M. ampla*, less pronounced in *M. neoampla*, and least in *M. multisetigera* (Gusev and Siver 2017). Following its description, the species has also been recorded from other localities in Vietnam, both in the northern (Gusev et al. 2021b) and central parts of the country (Gusev and Martynenko 2022).

In this study, we describe another taxon of the genus *Mallomonas* that combines features of fossil and modern species, present molecular data for all extant taxa, and establish the phylogenetic position of members within section Multisetigerae, including *M. multisetigera*, *M. neoampla*, and *M. rutabuliformis* sp. nov.

From a morphological point of view, *Mallomonas rutabuliformis* shares some features with *M. ampla* and *M. neoampla* and others with *M. multisetigera*. All taxa share similar papillae ornamentation on their scales and general scale morphology. *Mallomonas rutabuliformis* and *M. multisetigera* lack distinct anterior ribs, in contrast to *M. ampla* and *M. neoampla*, which exhibit well-developed anterior ribs. The scales of *M. rutabuliformis* (3.2–4.1 × 1.8–2.2 μm) are slightly larger than those of *M. multisetigera* cultures from Vietnam (2.3–3.5 × 1.2–1.7 μm) but smaller than those of both *M. neoampla* (4.9–7.4 × 2.5–4.1 μm) and *M. ampla* (4.7–6.7 × 3.1–4.1 μm). The V-rib on *Mallomonas rutabuliformis* scales is characteristically prominent and rounded, contrasting with the more acute-angled, hooded V-rib morphology observed in other members of the group.

The size and arrangement of the base plate pores differ among all four species, especially on the posterior flange, and represent a useful character for distinguishing among the taxa. *Mallomonas ampla* has small pores (30–55 nm) of similar diameter distributed more or less evenly across the entire scale, including up to six rows on the posterior flange (Siver and Lott 2012). The scales of *M. neoampla* also have small pores (30–40 nm) evenly distributed on the scale shield, but they are largely absent on the posterior flange. Based on TEM observations, the scales of *M. neoampla* exhibit circular patterns of less dense silica within the posterior flange. These patterns are similar in size to the large pores of *M. multisetigera* but are arranged similarly to the pores of *M. ampla*. However, SEM observations reveal that these less dense structures are clearly covered with silica on both sides of the scale. The base plate pores on *M. multisetigera* scales are of two distinct sizes, with notably larger pores (90–110 nm) on the posterior flange that are typically arranged in two concentric rows (Dürrschmidt 1982: figs 18, 19). *Mallomonas rutabuliformis* has small pores (10–30 nm) irregularly located on the basal plate, posterior flange, and dome. Circular patterns of less dense silica within the posterior flange are characteristic for this species, a feature also observed in *M. neoampla*. A rather interesting feature is their arrangement in a single row along the posterior rim, similar to that in *M. multisetigera*.

There are also differences in the structure of the bristles among the four species. The bristles of *M. multisetigera* are 2.6–5.0 µm long. The foot of the bristle is flattened and turned at an angle of approximately 45° or less relative to the shaft. While *M. rutabuliformis* exhibits similarly sized bristles (2.3–6.2 µm), its foot differs in being hook-shaped and angled at 45–90°, resembling the bristles of *M. ampla* and *M. neoampla*. All taxa share bifurcated distal ends, though with distinct configurations: *M. rutabuliformis* and *M. multisetigera* display acutely angled bifurcations, whereas *M. neoampla* presents a broader, rounded median part. Fossil specimens of *M. ampla* show damaged distal tips, preventing reliable assessment of their original structure.

The general structure of *Mallomonas rutabuliformis* scales also resembles that of *M. acidophila* (section Papillosae). The scales of *M. acidophila* possess a recessed, shallow dome (Gusev et al. 2023a). However, unlike in *M. rutabuliformis*, this dome is elongated in the transverse direction and can be oriented at various angles, whereas in the newly described species, the dome is rounded. Furthermore, the scales of *M. acidophila* are larger and lack pores on the basal plate and flanges.

Rather unexpected results were obtained when examining the phylogeny of representatives from this section using the SSU rDNA–*rbc*L dataset. All three studied species formed separate clades with long branches. However, they did not form a common monophyletic clade. Only *Mallomonas multisetigera* and *M. rutabuliformis*, which share similar scale sizes, clustered together in one clade. Nevertheless, the genetic distance between them was considerable. *Mallomonas neoampla* formed a separate clade with *M. vietnamica*, which has different morphology and, in our opinion, belongs to section Mallomonas, while, according to another view, it forms its own section, *Uncatusipedae* (Hao et al. 2024). Importantly, all four species—three from section Multisetigerae and one from section Mallomonas/*Uncatusipedae*—exhibit characteristics typical of fossil species, specifically an oval dome recessed from the distal scale margin, often angled relative to the longitudinal axis, and distinctive bristle morphology with a hook-shaped, flattened foot. The genetic distances between these species were also remarkably large. In our view, the long branches, reflecting a high number of nucleotide substitutions, prevent accurate determination of the phylogenetic positions of these species within the genus. This uncertainty arises from limited taxa sampling, lack of intermediate lineages, and insufficient data based on a small number of genes. At present, we conclude that morphotypes assigned to section Multisetigerae based on unequivocal similarities in scale ultrastructure represent distinct phylogenetic lineages. Two primary differences in scale morphology and ultrastructural elements can be discerned between these lineages. In *Mallomonas multisetigera* and *M. rutabuliformis*, the dome on the body scales is rounded or broadly oval, and the scale shape is more or less symmetrical. In contrast, *M. neoampla*—which, together with *M. vietnamica*, forms a separate phylogenetic clade—possesses an elongated oval dome positioned at a varying angle to the cell’s longitudinal axis and a distinctly asymmetrical scale shape. Therefore, additional comprehensive studies of this group are needed.

## Supplementary Material

XML Treatment for Mallomonas rutabuliformis

XML Treatment for Mallomonas multisetigera

## ﻿Additional information

### Use of AI

No use of AI was reported.

### Author ORCIDs

Evgeniy S. Gusev https://orcid.org/0000-0002-7397-5808

Tatiana V. Safronova https://orcid.org/0000-0003-4098-5927

Yulia А. Podunay https://orcid.org/0000-0002-0519-2908

Hoan Tran https://orcid.org/0000-0002-0143-9918

Alma B. Mohagan https://orcid.org/0000-0002-8303-5131

Nikita A. Martynenko https://orcid.org/0000-0002-7286-003X

### Data availability

All of the data that support the findings of this study are available in the main text.
